# The relationship between clinicobiochemical markers and depression in women with polycystic ovary syndrome

**Published:** 2014-12

**Authors:** Mohammad Ehsan Rahiminejad, Amirhossein Moaddab, Soghra Rabiee, Farzaneh Esna-Ashari, Shiva Borzouei, Seyyed Mohammad Hosseini

**Affiliations:** 1 *Research Center of Behavioral Sciences and Dependency, Hamedan University of Medical Sciences, Hamedan, Iran.*; 2 *Department of Obstetrics and Gynecology, Baylor College of Medicine, Houston, TX, USA.*; 3 *Department of Obstetrics and Gynecology, Hamedan University of Medical Sciences, Hamedan, Iran.*; 4 *Department of Community Medicine, Hamedan University of Medical Sciences, Hamedan, Iran.*; 5 *Department of Internal Medicine, Hamedan University of Medical Sciences, Hamedan, Iran.*

**Keywords:** *Polycystic ovary syndrome*, *Depression*, *Obesity*, *Hirsutism*, *Complication*

## Abstract

**Background:**

Previous studies have demonstrated that clinical features of Polycystic ovary syndrome (PCOS) are associated with a lower degree of health, self, and sex satisfaction.

**Objective:**

Our study aimed to investigate possible associations between depression and different clinicobiochemical markers of PCOS.

**Materials and Methods:**

In a cross-sectional analytic study, 120 PCOS women aged 18-45 yr, were enrolled. Beck Depression Inventory was used to assess depression. Also, all participants underwent biochemical studies. Individuals with 15 points and more in Beck test were referred to a psychiatrist to participate in a complementary interview for the diagnosis of depression based on Diagnostic and Statistical Manual of Mental Disorders IV (DSMIV-TR) criteria.

**Results:**

Among the study participants, 82 women (68.3%) were non-depressed, and 38 patients (31.7%) had some degrees of depression. According to the psychiatric interview, 10 patients (8.3%) had major depression, 22 patients (18.3%) had minor depression and 6 patients (5%) had dysthymia. We failed to show any significant difference in body mass index, hirsutism, infertility, serum total testosterone, lipid profile, and the homeostasis model assessment of insulin resistance (HOMA-IR) between depressed and non-depressed subjects (p>0.05). Using Spearman correlation, we did not find a positive correlation between BDI scores and clinicobiochemical markers for all PCOS subjects (-0.139≤r≤+0.121, p>0.05).

**Conclusion:**

In spite of high rate of depression in women with PCOS, there was no significant association between Clinicobiochemical Markers and depression.

## Introduction

Polycystic ovary syndrome (PCOS) is the most common endocrine disorder among women of reproductive age, affecting 4-18% of these women (1, 2). It has significant clinical and biochemical sequelae, including reproductive features (such as infertility, hyperandrogenism, and hirsutism) and metabolic derangements (such as obesity, insulin resistance, impaired glucose tolerance, type II diabetes mellitus, and adverse cardiovascular risk profiles) (3). 

In addition, women with PCOS have a greater degree of mood dysfunction, psychiatric disorders and emotional distress than women without PCOS (4, 5). A recent systematic review and meta-analysis of 12 published comparative studies between women with PCOS and healthy controls revealed higher depression and anxiety scores in patients with PCOS, is associated with Body Mass Index (BMI) (6). 

In addition, previous studies have demonstrated that the clinical features associated with PCOS, such as physical appearance (hirsutism, obesity and acne), menstrual abnormalities (oligomenorrhoea) and infertility are associated with a lower degree of satisfaction with health, self, and sex affecting Health Related Quality of Life (HRQoL) (7, 8). Despite this proved issue, the association between infertility, biochemical hyperandrogenism, hirsutism, acne, body image and depression is still controversial (9). Depression is a major public health problem in both developed and developing countries and a leading cause of disability and global disease burden (10, 11). 

Surveys in the United States have shown that major depressive disorder (MDD) based on the Diagnostic and Statistical Manual IV (DSM-IV) affects approximately 14.8 million American adults or about 12-14% of the 18-44 year-old American women in a given year (12, 13). Furthermore, it is well known that MDD is more prevalent in women than in men (14). In Iran, based on a systematic review the prevalence of MDD among Iranian adults is 4.1% and women are more likely to have MDD, approximately 1.95 times more than men (15). In the literature, depression in women with PCOS has a wide range of prevalence rates, reported between 14% and 64% (16). In Shakerardekani's study the prevalence of depression in PCOS women was 45% according to Beck Depression Inventory (BDI) (17). It seems that social and cultural differences and various assessment methods have led to this wide range of prevalence rates. This shows the necessity for further studies in different ethnic groups worldwide (16).

The aim of this study was to investigate the rate of depression among women with PCOS, and to analyze associations between depression and different clinicobiochemical markers of PCOS.

## Results

Among all participants, 82 (68.3%) patients were non-depressed, and 38 (31.7%) patients had various degrees of depression. Based on the Beck Inventory scores, 28 (23.3%) patients suffered from mild depression, 9 (7.5%) had moderate depression, and one patient (0.8%) had severe depression. The mean age of individuals in the non-depressed and depressed patients were 23.6±4.7 and 24.8±4.6 year, respectively, with no significant differences (p=0.192). Based on psychiatric interview, 10 patients (8.3%) had MDD, 22 (18.3%) had minor depression and 6 patients (5%) suffered from dysthymia. There was no difference in the frequency of depression relative to marital status. Of the 82 non-depressed patients 41 (50%) were married and 41 (50%) were single. Of 38 depressed patients 17 (44.7%) were married and 21 (51.3%) were single (p=0.591). 

The mean BMI in non-depressed patients was 24.8±4.3, which was not significantly different from that in depressed patients (24.96±5.01) (p=0.177). The assessment for hirsutism showed that among 38 depressed patients, 23 (60.5%) and of 82 patients without depression 38 patients (46.3%) had hirsutism. The present study failed to show any significant differences in serum TT, TG, total cholesterol, LDL, HDL and HOMA-IR between depressed and non-depressed patients. 

In addition, these measurements revealed no differences between patients with mild and moderate depression. Table I presents a comparison of the clinical and biochemical parameters among depressed and non-depressed patients. Investigation of infertility in patients showed that 28 (34.1%) of non-depressed patients suffered from infertility, 11 (13.4 %) had been fertilized and 43 (52.5%) had unknown fertility status. Among depressed patients, 7 (18.4%), 8 (21.1%) and 23 (60.5%) patients had infertility, had been fertilized and had unknown fertility status, respectively.

No differences were found in infertility prevalence based on depression status in women with PCOS. All the individuals were asked about their main concern for health issues related to their disease; 42 patients (35%) considered hirsutism as their most important concern, while 27 patients (22.5%) and 44 patients (36.7%) believed obesity and infertility were their main concerns, respectively. Using Spearman correlation, we did not find a positive correlation between BDI scores and clinicobiochemical markers for all PCOS subjects (-0.139≤r≤+0.121, p>0.05) (Table II).

**Table I:** Comparison of demographic and clinicobiochemical markers between depressed and non-depressed patients based on Beck Inventory scores

		**No depression (n=82)**	**Depression (n=38)**	**p-value**
Beck		7.89 ± 4.34	25.45 ± 8.83	
Age (yr)		23.6 ± 4.7	24.8 ± 4.6	0.192^a^
Testosterone (ng/mL)		0.66 ± 0.18	0.73 ± 0.16	0.634^a^
TG (mg/dl)		132.70 ± 59.96	136.89 ± 49.81	0.739^a^
LDL (mg/dL)		106.54 ± 21.97	112.58 ± 23.50	0.554 a
HDL (mg/dL)		44.11 ± 6.95	44.11 ± 6.54	0.359 a
Cholesterol (mg/dL)		170.37 ± 21.66	174.63 ± 22.91	0.366 a
HOMA-IR		1.40 ± 0.53	1.34 ± 0.51	0.746 a
BMI (kg/m^2^)		24.8 ± 4.3	24.9 ± 5.1	0.177 a
Marital status				
	Married	41 (50%)	17 (44.7%)	0.591 b
	Single	41 (50%)	21 (55.3%)	
Infertile				
	Yes	28 (34.1%	7 (18.4%)	0.177 b
	No	11 (13.4%)	8 (21.1%)	
	Unknown	43 (52.5%)	23 (60.5%)	
Hirsutism				
	Yes	38 (46.3%)	23 (60.5%)	0.148 b
	No	44 (53.7%)	15 (39.5%)	

TG: triglycerides

LDL: low-density lipoprotein

HDL: high-density lipoprotein

BMI: Body Mass Index

HOMA-IR: homeostasis model assessment of insulin resistance ( fasting glucose (μU/L)× fasting insulin (mmol/L)/22.5)

Hirsutism (Modified Ferriman-Galwey score ≥ 6)

a Student’s* t* test

b chi-squared test

**Table II:** Spearman correlations between BDI and clinicobiochemical markers of PCOS

		**Testosterone**	**LDL**	**TG**	**HDL**	**Cholesterol**	**BMI**	**HOMA-IR**
BDI Score								
	Correlation Coefficient	0.121	0.108	0.072	0.032	0.064	0.012	-0.139
	Sig. (2-tailed) *	0.189	0.239	0.435	0.731	0.485	0.899	0.131

TG: triglycerides

LDL: low-density lipoprotein

HDL: high-density lipoprotein

BMI: Body Mass Index

BDI: Beck Depression Inventory

HOMA-IR: homeostasis model assessment of insulin resistance

* Correlation is significant at the 0.05 level (2-tailed).

**Figure 1:**
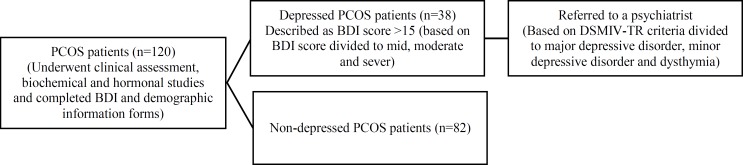
Flow diagram of patients through the study protocol.

## Discussion

The present study was performed to assess depression in patients with PCOS. We aimed to describe the signs and symptoms of PCOS differences between depressed and non-depressed subjects, respectively. In the present study, the rate of MDD was 8.3%, which is lower than that in other studies (6, 21, 22). On the other hand, the overall rate of depression in our study was lower than previous study in Iran measured, by a similar method (31.7% vs. 45%) (17). Although these results show that the rate of MDD among PCOS patients with MDD in the general population has more than doubled. (4.1% vs. 8.3%) (15). This result suggests that attention to psychological aspects should be considered in PCOS patients.

**Depression and clinical manifestations of PCOS**

Obesity, especially abdominal obesity, is a common characteristic of these patients, which compromises the patient's appearance and results in many esthetic problems for them (23). However, despite the variety of information about the impact of obesity on psychological stress and depression, in the present study the mean BMI did not differ in non-depressed and depressed patients. Our findings do not support the previous studies that considered obesity as a risk factor for depression in patients with PCOS (24, 25). Along with this study, some studies found no relationship between depression and obesity in patients with PCOS (6, 22). In the present study, hirsutism, as a clinical manifestation of PCOS, was not significantly different in depressed and non-depressed patients, inconsistent with some previous studies which considered hirsutism concomitant with higher grade of depression (26). 

In this study, infertility rate was 29.2%, and there was no significant association between infertility and depression in participants. It is worth mentioning that a great number of patients (55%) were not aware of their own ability to conceive, which might have resulted in misjudgment. Consistent with this study, Hollinrake *et al* found that on analysis of the PCOS group alone, similar numbers of depressed and non-depressed PCOS women reported a history of infertility (20). We found that PCOS patients considered infertility as the most important concern (36.7%). Since infertility treatment in these patients is feasible, with a systematic treatment and reassurance, the patient can reduce much of the concern.

**Depression and biochemical markers of PCOS**

To evaluate differences between depressed and non-depressed PCOS patients, we measured biochemical markers and compared them between the two groups. The relationship between depression and insulin resistance, as one of the leading factors in the pathophysiology of PCOS, has been examined in a few studies which have shown conflicting results (26-28). Depression is associated with increased cortisol levels, increased sympathetic nervous system activity and decreased serotonin in the central nervous system. These features are also associated with insulin resistance (27). 

If depression affects insulin resistance, people with insulin resistance (e.g. patients with PCOS), who simultaneously suffer from depression, are exposed to metabolic condition changes which results in type II diabetic development. Timely and robust treatment of depression may be a good and appropriate strategy to prevent or reduce development of insulin resistance (27, 28). We found no significant differences in insulin resistance between the two groups. However, it seems that evaluation of relationship between insulin resistance and psychological stress in patients with PCOS needs to be investigated further.

Women with PCOS may have clinical presentations or biochemical evidence of hyperandrogenism. Several studies have shown a correlation between the severity of depression and serum androgens (29-31). Despite absence of significant differences between the two groups, in this study the mean serum testosterone level in patients with depression was slightly higher than non-depressed patients (0.74 vs.0.67). The results of the present study are similar to those reported by Hollinrake *et al* (20). Lipid profiles, including total cholesterol, HDL, LDL and TG, were also compared and no differences were found between depressed and non-depressed PCOS patients.

## Conclusion

In spite of high rate of depression in women with PCOS, we failed to show an association between any of the clinicobiochemical markers and depression. However, like Farrell and Antoni, we emphasize that the physiological and psychological factors among women with PCOS are strongly interrelated and suggest that in medical management of PCOS both physiological and psychological approaches should be used (4).
